# Echocardiographic assessment of brain sparing in small-for-gestational age infants and association with neonatal outcomes

**DOI:** 10.1038/s41598-023-37376-7

**Published:** 2023-06-23

**Authors:** Ju Ae Shin, Jae Young Lee, Sook Kyung Yum

**Affiliations:** Department of Pediatrics, Seoul St. Mary’s Hospital, College of Medicine, The Catholic University of Korea, 222 Banpo-Daero, Seocho-Gu, Seoul, 06591 Republic of Korea

**Keywords:** Medical research, Risk factors

## Abstract

Brain sparing is an adaptive phenomenon (redistribution of blood flow to the brain) observed in fetuses exposed to chronic hypoxia, who are at risk of intrauterine growth restriction. Here, we assessed the blood flow distribution during the early neonatal period (< 7 days of life) using echocardiography, and evaluated the impact of brain-sparing on postnatal course and neurodevelopmental outcomes. This retrospective study included 42 small-for-gestational age (SGA) infants [further classified into asymmetric SGA (a-SGA, n = 21) and symmetric SGA (s-SGA, n = 21) groups according to their birth head circumference percentiles], and 1: 2 matched appropriate-for-gestational age (AGA) infants (n = 84) admitted to the neonatal intensive care unit. Left ventricular (LV) stroke volume, LV cardiac output (LVCO), upper body blood flow (UBBF), and UBBF/LVCO ratio (%) were significantly higher in both a-SGA and s-SGA infants than in AGA infants. Both a-SGA and s-SGA groups consisted predominantly of infants with higher UBBF/LVCO (%). A UBBF/LVCO ≥ 58.2% (3rd interquartile range) was associated with a later need for rehabilitative therapy after discharge. In summary, brain-sparing effect may continue during the early postnatal life in SGA infants, and may be a promising marker to detect future adverse neurodevelopmental outcomes.

## Introduction

Fetal growth restriction (FGR) is a condition in which fetal growth is pathologically reduced. The etiologies of FGR include chronic hypoxia caused by maternal hypertensive disorder, placental insufficiency, and disproportionate placental perfusion in twin pregnancies^1^. The affected infants redistribute cardiac output in preference for essential organs–in particular, the brain. This phenomenon is known as brain sparing^2^. In fetal circulation, most of the right ventricular blood is directed towards the ductus arteriosus and into the descending aorta, while oxygenated and nutrient-rich umbilical vein blood flows through the foramen ovale, into the left ventricle, from which most of the output is directed to the upper body including upper limbs and brain. In an FGR setting, peripheral vascular beds are vasoconstricted, leading to increased right ventricle afterload; conversely, cerebral arteries are vasodilated, leading to a decreased left ventricle afterload, thus, increasing the blood supply to the brain^3^. Brain sparing is believed to be an adaptive mechanism of growth-restricted fetuses for maintaining sufficient oxygen and nutrient flow to ensure adequate brain growth and development. However, studies have reported controversial results regarding whether this adaptive process is a fully protective mechanism^4–6^.

In the literature, various antenatal [e.g., the middle cerebral artery (MCA) pulsatility index (PI)^7^, cerebro-placental ratio (CPR)^8^] and postnatal parameters [e.g., MCA or anterior cerebral artery (ACA) blood flow velocity or PI^9^] have been used to diagnose brain sparing. In addition, near-infrared spectroscopy^10,11^ was used to check the possible postnatal brain-sparing effect. However, these methods represent indirect measurements of blood flow, resulting in variable associations between brain-sparing and neurodevelopmental outcomes.

Considering that only a small fraction of cardiac output flows into the upper limbs and chest, we deemed that the upper body blood flow (UBBF), which is obtained by subtracting the descending aorta blood flow from the left ventricular cardiac output (LVCO) based on postnatal echocardiography, can be taken as a surrogate for brain blood flow, as described previously^12,13^.

Here, we aimed to evaluate the brain-sparing effect and its association with neonatal outcomes using direct measurements obtained via echocardiography in growth-restricted infants. We hypothesized that the brain-sparing effect would be sustained during the early postnatal days in growth-restricted infants and that such findings would be associated with adverse in-hospital and post-discharge neonatal outcomes.

## Materials and methods

### Study population

This retrospective study included infants admitted to the neonatal intensive care unit (NICU) of our institute between September 2015 and August 2021. Additionally, the infants had to have echocardiographic images obtained within 7 days of life. The institutional review board of Seoul St. Mary’s Hospital (approval number: KC22RISI0635) approved the study design and waived the need for informed consent, owing to the retrospective nature of the study. All study methods were carried out in accordance with relevant guidelines and regulations. Only infants born at ≥ 32 weeks' gestation were included, because very preterm infants born before this threshold exhibit distinct cardiopulmonary physiology from those born later^14^ and tend to have a significantly higher prevalence of health complications, including patent ductus arteriosus (PDA), which can affect echocardiographic data and neonatal outcomes^15^. The following exclusion criteria were set: infants with major congenital anomaly, chromosomal or genetic anomaly, newborns with persistent pulmonary hypertension, hemodynamically significant PDA necessitating medical or surgical treatment, moderate to severe hypoxic-ischemic encephalopathy undergoing therapeutic hypothermia, intrauterine infection or early onset sepsis, arrhythmia, and other hemodynamically compromised conditions based on medical record review. The typical reasons for performing echocardiography in the included infants were as follows: to rule out cardiac problems in cases with sustained tachypnea despite respiratory support, cardiomegaly on the chest radiograph, routine screening in moderate-to-late preterm infants, infants born to mothers with diabetes, or apnea/desaturation during feeding.

### Definition of and stratification according to growth pattern at birth

Small-for-gestational age (SGA) infants who satisfied the inclusion criteria were selected to comprise the study group. SGA was defined as birth- weight below the 10th percentile for the specific gestational age based on the Korean Newborn Survey and Statistics^16^. For each of the included SGA infants, appropriate-for-gestational age (AGA, birth weight percentile ≥ 10th and < 90th percentile) infants were matched to serve as the control group at a 1:2 ratio for the following basic characteristics: gestational age, sex, and postnatal age when echocardiography was performed. Gestational age was categorized into four groups (32–34, 35–36, 37–38, and 39–41 weeks) for matching.

### Data collection

#### Baseline demographic characteristics

Gestational age, birthweight, length and head circumference at birth, sex, mode of delivery, 1-min and 5-min Apgar scores, infant plurality (singleton or multiple births), maternal history and perinatal morbidities, blood gas analysis results, blood pressure, heart rate, percutaneous oxygen saturation (SpO_2_), and requirement for respiratory support at the time of echocardiography were determined by reviewing medical records.

#### Echocardiographic data

**Figure 1:**
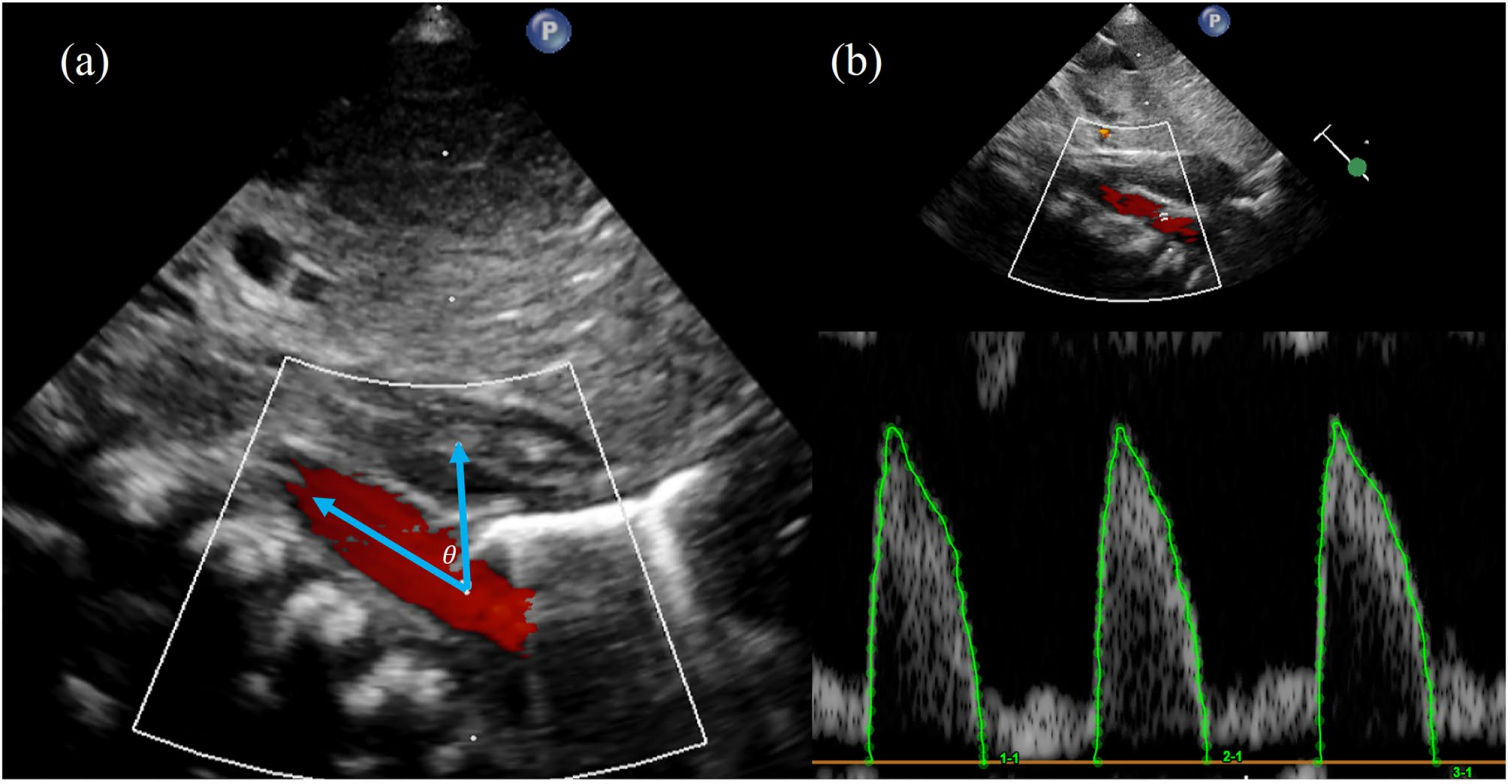
Echocardiographic images for obtaining descending aorta blood flow. (**a**) A subcostal sagittal view showing descending aorta (Ao) blood flow at the level of diaphragm (D). Two arrows indicate the directions of blood flow (left) and ultrasound beam (right) with an angle of *θ*. (**b**) The VTI was obtained by tracing pulsed-wave Doppler image and divided by cos *θ* for angle correction between blood flow and ultrasound beam. Abbreviations *VTI* Velocity time integral.

Postnatal cardiac structure and functions were assessed during the early neonatal period (< 7 days of life) by two experienced pediatric cardiologists, using an Affiniti 50 echocardiography system (Philips Ultrasound, Bothell, Seattle, WA) with a pediatric cardiac and neonatal head probe (12–14 MHz). Echocardiographic studies were performed during natural sleep or in quiet and alert states. Echocardiographic measurement of blood flow across a target vessel was performed by multiplying the cross-sectional area (CSA) of the vessel and the velocity time integral (VTI) of pulsed-wave Doppler at a specific point, and heart rate^17^. Left ventricular cardiac output (LVCO) was calculated by multiplying the left ventricle stroke volume (LVSV) and heart rate. LVSV was derived by multiplying the VTI of the aortic valve pulsed-wave Doppler and the CSA of the aortic valve. The CSA of the aortic valve was obtained by measuring the diameter at the hinge point of the aortic valve annulus at end-systole at the parasternal long-axis view. The VTI of the aortic valve was obtained using pulsed-wave Doppler at the apical five-chamber view. Descending aorta blood flow (DABF) was derived by multiplying the pulsed-wave Doppler VTI and CSA of the descending aorta at the level of the diaphragm in a low subcostal sagittal view, and heart rate. For obtaining pulsed wave Doppler VTI of the descending aorta for calculating DABF, an appropriate Doppler angle correction was applied (Fig. 1). The upper body blood flow (UBBF) was obtained by subtracting the DABF from the LVCO^12,13^. The measurements were obtained from three consecutive cardiac cycles and averaged. A pediatric cardiologist (J.A.S.) with four years of experience reviewed the archived echocardiographic images and obtained these values using DCASi version 1.3. Figure 1 illustrates an example of echocardiographic parameter obtainment.

#### Neonatal outcomes

Brain imaging, such as brain sonography or magnetic resonance imaging (MRI), anti-epileptic drug (AED) administration, NICU admission duration, and time required for full feeding were collected for in-hospital outcome analysis. Measurements and growth percentiles for head circumference, weight, and length at 4–5 months of corrected age were obtained as post-discharge outcomes. Percentiles for weight, head circumference, and length at the post-discharge time point were obtained from the growth curve based on the Korean Newborn Survey and Statistics^18^.

Neurodevelopmental assessment of infants at 4–5 months of corrected age was performed using the Korean Developmental Screening Test for Infants and Children (K-DST), developed in September 2014 by the Korean Pediatric Society and presided over by the Korea Center for Disease Control and Prevention^19^. The K-DST consists of questionnaires for five categories (gross motor function, fine motor function, cognition, language, and social interaction). The summed score for each category was classified according to the following four levels: higher-than-peer level (≥ 1 standard deviation [SD]), peer level (< 1SD and ≥ 1SD), follow-up evaluation is recommended (< − 1SD and ≥  − 2SD), and detailed evaluation is warranted (< − 2SD). If the score for any one of the five categories was <  − 2SD, the infant was considered “screen positive” for the K-DST, which implied a suspicion of developmental delay and required that the infant be further evaluated. In addition to the K-DST results, we reviewed whether the infants had received rehabilitative therapy at any time after discharge until the last follow-up visit at our hospital.

### Statistical analysis

SPSS version 22 (SPSS Inc., Chicago, Illinois, USA) was used for the statistical analysis. For analysis, the study group was further stratified as “asymmetric” SGA (a-SGA) when only the birthweight was below the 10th percentile and “symmetric” SGA (s-SGA) when both the birthweight and head circumference were below the 10th percentile. Although it is not usual to determine whether SGA is symmetric or asymmetric, we adopted these terms in the current study to discretely evaluate the implications of brain sparing. Baseline characteristics, echocardiographic parameters, and in-hospital and post-discharge neonatal outcomes were compared among a-SGA, s-SGA, and matched AGA infants. Specifically, we investigated whether the brain-sparing effect of growth-restricted neonates continued after birth by measuring left ventricular cardiac output (LVCO) and upper body blood flow (UBBF). Greater UBBF, which implies blood flow redistribution, was used as a surrogate for brain sparing in the present study.

We also analyzed neonatal outcomes according to the parameters implying brain sparing. Comparisons between groups were assessed using one-way ANOVA to analyze parametric continuous variables, and the Kruskal–Wallis test was used to analyze non-parametric continuous variables. Categorical variables were analyzed using chi-square and Fisher's exact tests, as appropriate. *P* value < 0.05 was considered significant. Post hoc analysis using the Bonferroni method was performed when categorical variables were analyzed for the three groups. Receiver operating characteristic (ROC) analysis was used to determine whether UBBF and UBBF/LVCO were significantly associated with unfavorable neonatal outcomes.

## Results

The baseline characteristics of the included infants and between-group comparisons are shown in Table 1. A total of 42 SGA (each 21 for a-SGA and s-SGA) and 84 AGA neonates were enrolled. The main disease status/reason for echocardiogram were as follows: (1) prematurity and/or LBW (n = 26), (2) screening in infants with maternal diabetes (n = 12), (3) cardiac murmur (n = 17), (4) tachypnea or intermittent desaturation (n = 30), (5) enlarged or abnormal cardiac silhouette on chest radiograph (n = 20), (6) others, including screening for single umbilical artery, prenatal suspicion for ventricular septal defect or coarctation of the aorta (but proved to be normal after postnatal echocardiogram), pallor or mild cyanotic appearance, etc. (n = 21). The mean ± SD head circumference was significantly smaller in s-SGA infants (30.1 ± 1.3 cm) than in AGA (33.4 ± 1.7 cm) and a-SGA (33.0 ± 3.1 cm) infants. The 1-min Apgar score was lowest in s-SGA infants (median, 6). The s-SGA infants showed a higher prevalence of maternal hypertension than AGA and SGA infants. Oligohydramnios was more prevalent in both a-SGA and s-SGA infants than in AGA infants; however, the difference was not statistically significant. Other characteristics were similar between the groups.

**Table 1:** Baseline characteristics of AGA, a-SGA, and s-SGA groups.

	AGA (N = 84)	a-SGA (N = 21)	s-SGA (N = 21)	*p*
Gestational age (weeks)	36.9 [34.3–38.6]	37.3[34.7–39.0]	35.4 [34.2–37.3]	NS
Birthweight (g)	2,838 [2,248–3,293]	2,260 [1,892–2,510]	1,970 [1,565–2,135]	< 0.001^a,b^
Birthweight < 10th percentile	0 (0.0)	21 (100.0)	21 (100.0)	< 0.001^a,b^
Length at birth (cm)	48.0 [45.4–50.3]	46.0 [41.9–47.5]	42.0 [40.2–44.0]	< 0.001^a,b^
Length < 10th percentile	10 (11.9)	12 (57.1)	17 (81.0)	< 0.001^a,b^
Head circumference at birth (cm)	33.4 ± 1.7	33.0 ± 3.1	30.1 ± 1.3	< 0.001^b,c^
Head circumference < 10th percentile	1 (1.2%)	0 (0.0%)	21 (100.0)	< 0.001^b,c^
Male sex	46 (54.8)	10 (47.6)	11 (52.4)	NS
Cesarean delivery	65 (77.4)	18 (85.7)	19 (90.5)	NS
Multiple births	16 (19.0)	4 (19.0)	8 (38.1)	NS
1-min Apgar	7[6–9]	8[4–9]	6[4–7]	0.035^c^
5-min Apgar	9[7–10]	9[7–10]	8[7–9]	NS
Maternal age (years)				NS
Maternal diabetes	17 (20.2)	3 (14.3)	2 (9.5)	NS
Maternal hypertension	8 (9.5)	1 (4.8)	7 (33.3)	0.009*^b,c^
Oligohydramnios	2 (2.4)	3 (14.3)	3 (14.3)	0.054*
Blood gas analysis at birth				
pH	7.33 ± 0.09	7.32 ± 0.07	7.31 ± 0.08	NS
pCO_2_	42.9 ± 11.1	41.4 ± 7.0	46.0 ± 12.9	NS
Base deficit	3.9 [2.1–5.2]	4.0 [2.8–6.1]	4 [1.8–5.5]	NS

a. *p* < 0.05, AGA vs. a-SGA; b. *p* < 0.05, AGA vs. s-SGA; c. *p* < 0.05, a-SGA vs. s-SGA.

*Fisher’s exact test.

*AGA* Appropriate for gestational age, *a-SGA* Asymmetric small-for-gestational age, *CO*_*2*_, Carbon dioxide, *s-SGA* Symmetric small-for-gestational age.

The median postnatal age at echocardiography was 2–3 days depending on the patient group (Table 2). LVSV, LVCO, UBBF, and UBBF/LVCO were significantly increased in both the a-SGA and s-SGA infants when compared to the AGA infants, while no significant difference between the s-SGA and a-SGA groups was observed. In particular, mean ± SD UBBF/LVCO (%) was very similar in the a-SGA (56.6 ± 14.7) and s-SGA infants (56.6 ± 14.1), in contrast to the AGA (42.0 ± 11.9) infants. The characteristics of respiratory support and supplemental oxygen, sedative use, and inotropic agent(s) use were not significantly different between growth pattern-stratified groups. No statistically significant differences were noted in the blood gas analysis results or blood pressure. The mean values of hemoglobin, hematocrit, and heart rate were higher in the a-SGA and s-SGA infants than in the AGA infants, but the difference was not statistically significant.

**Table 2:** Neonatal characteristics at echocardiographic assessment and echocardiographic parameters representing brain-sparing patterns.

	AGA (N = 84)	a-SGA (N = 21)	s-SGA (N = 21)	*p*
Initial echocardiographic assessment				
PNA (days)	3[2–4]	2[2–4]	3[2–4]	NS
Respiratory support	66 (78.6%)	14 (66.7)	13 (61.9)	NS
Invasive MV†	25 (37.9)	6 (42.9)	6 (46.2)	
Noninvasive MV†	41 (62.1)	8 (57.1)	7 (53.8)	
Supplemental oxygen‡	6 (33.3)	2 (28.6)	1 (12.5)	NS
Sedative use	10 (11.9)	4 (19.0)	1 (4.8)	NS
Inotrope(s) use	7 (8.3)	2 (9.5)	1 (4.8)	NS
Heart rate (BPM)	141 ± 21	144 ± 22	151 ± 17	NS
Systolic BP (mmHg)	69[64–73]	71[62–72]	67[63–71]	NS
Diastolic BP (mmHg)	43 ± 6	43 ± 7	41 ± 4	NS
Blood pH	7.35 ± 0.07	7.38 ± 0.09	7.35 ± 0.08	NS
Blood pCO_2_ (mmHg)	44.6 ± 11.0	39.9 ± 11.8	45.3 ± 13.6	NS
Blood base deficit	1.3 ± 3.3	1.7 ± 3.6	1.3 ± 3.5	NS
Hemoglobin (g/dL)	16.7 ± 2.6	17.2 ± 3.7	17.6 ± 1.8	NS
Hematocrit (%)	48.9 ± 7.7	51.6 ± 9.0	51.5 ± 5.0	NS
PDA present	37 (44.0)	7 (33.3)	10 (47.6)	NS
PDA size (mm)	0.5[0.5–2.0]	1.5[0.5–2.0]	1.8[0.5–2.1]	NS
LV stroke volume (ml/kg)	1.32 ± 0.29	1.58 ± 0.26	1.73 ± 0.43	< 0.001^a,b^
LVCO (ml/kg/min)	186 [151–210]	224 [196–259]	271 [217–298]	< 0.001^a,b^
DABF (ml/kg/min)	103[84–118]	92[74–111]	106[80–131]	NS
UBBF (ml/kg/min)	73 [55–99]	132 [93–154]	155 [116–190]	< 0.001^a,b^
UBBF/LVCO (%)	42.0 ± 11.9	56.6 ± 14.7	56.6 ± 14.1	< 0.001^a,b^

a. *p* < 0.05, AGA vs. a-SGA; b. *p* < 0.05, AGA vs. s-SGA; c. *p* < 0.05, a-SGA vs. s-SGA.

^†^Analyzed in infants with respiratory support. ‡ Analyzed in infants without respiratory support.

*AGA* Appropriate for gestational age, *a-SGA* Asymmetric small-for-gestational age, *BP* Blood pressure, *BPM* Beats per minute, *CO*_*2*_ Carbon dioxide, *DABF* Descending aorta blood flow, *LV* Left ventricle, *LVCO* Left ventricle cardiac output, *MV* Mechanical ventilation, *PDA* Patent ductus arteriosus, *PNA* Postnatal age, *s-SGA* Symmetric small-for-gestational age, *UBBF* Upper body blood flow.

Table 3 compares the in-hospital outcomes, post-discharge growth, and neurodevelopmental outcomes at a corrected age of 4–5 months for AGA, a-SGA, and s-SGA groups. There were no significant differences in brain imaging findings, AED administration, hospital stay length, or a postnatal day at full feeding. At a corrected age of 4–5 months, body weight, length, and head circumference were greater in AGA infants than in a-SGA and s-SGA infants. The proportion of infants whose weight, length, and head circumference were below the 10th percentile was different among the three groups. In particular, although the mean ± SD head circumference was significantly smaller for a-SGA (41.3 ± 1.6) and s-SGA (41.2 ± 1.7) infants than for AGA infants (42.4 ± 1.6), the proportion of infants with head circumference < 10th percentile was significantly different only between the AGA (5.3%) and a-SGA (41.2%) groups (*p* < 0.001). Regarding the K-DST results, the proportion of screen-positive patients with a score < -2SD in any field was highest in the s-SGA infants (55.6%), although statistical significance was found only in comparison with the AGA infants (17.1%). When analyzed within specific fields, a significantly greater proportion of infants in the a-SGA and s-SGA groups screened positive for the gross motor field compared to those in the AGA group (29.4% *vs*. 50.0% *vs*. 7.9%, respectively), while the greatest proportion of infants in the s-SGA group (22.2%) screened positive in the cognitive field (2.6% in the AGA and 5.9% in the a-SGA infants). The proportion of patients who received rehabilitative therapy at any point during the follow-up period after discharge was significantly higher than that of AGA infants (21.3% vs. 58.8% vs. 66.7%, respectively).

**Table 3:** In-hospital and post-discharge outcomes of AGA, a-SGA, and s-SGA groups.

	AGA (N = 84)	a-SGA (N = 21)	s-SGA (N = 21)	*p*
In- hospital outcomes				
Abnormal brain imaging findings†	27 (41.5)	10 (55.6)	10 (55.6)	NS
GMH/IVH	25 (38.5)	7 (38.9)	9 (50.0)	NS
PVL	2 (3.1)	1 (5.6)	0 (0.0)	NS
AED administration	5 (6.0)	0 (0.0)	0 (0.0)	NS
PNA of full feeding achievement (days)	6[4–9]	8[6–9]	7[6–12]	NS
PNA at discharge (days)	13[10–18]	16[12–29]	11[18–35]	NS
Post-discharge outcomes				
At corrected age 4–5 months	(N = 76)	(N = 17)	(N = 18)	
Body weight (kg)	7.7 ± 0.8	6.9 ± 0.8	6.6 ± 0.9	< 0.001^a,b^
Body weight < 10th percentile	1 (1.3)	2 (11.8)	5 (27.8)	< 0.001^b^
Length (cm)	65.4 ± 2.6	62.1 ± 3.1	62.1 ± 3.4	< 0.001^a,b^
Length percentile < 10th percentile	2 (2.6)	5 (29.4)	6 (33.3)	< 0.001^a,b^
Head circumference (cm)	42.4 ± 1.6	41.3 ± 1.6	41.2 ± 1.7	< 0.003^a,b^
Head circumference < 10th percentile	4 (5.3)	7 (41.2)	4 (22.2)	< 0.001^a^
< − 2SD in at least 1 field of K-DST	13 (17.1)	6 (35.3)	10 (55.6)	0.002^b^
Gross motor	6 (7.9)	5 (29.4)	9 (50.0)	< 0.001^a,b^
Fine motor	2 (2.6)	2 (11.8)	3 (16.7)	NS
Cognitive	2 (2.6)	1 (5.9)	4 (22.2)	0.009^b^
Language	4 (5.3)	1 (5.9)	1 (5.6)	NS
Social	5 (6.6)	1 (5.9)	3 (16.7)	NS
RT at any point after discharge	16(21.3)	10(58.8)	12(66.7)	< 0.001^a,b^

a*. p* < 0.05, AGA vs. a-SGA; b. *p* < 0.05, AGA vs. s-SGA; c. *p* < 0.05, a-SGA vs. s-SGA.

^†^ Analyzed in infants with brain imaging results. (65 AGA, 18 a-SGA, and 18 s-SGA infants).

*AED* Antiepileptic drug, *AGA* Appropriate for gestational age, *a-SGA* asymmetric small-for-gestational age, *GMH* Germinal matrix hemorrhage, *IVH* Intraventricular hemorrhage, *K-DST* Korean developmental screening test for infants and children, *PNA* Postnatal age, *PVL* Periventricular leukomalacia, *RT* Rehabilitative therapy, *SD* Standard deviation, *s-SGA* Symmetric small-for-gestational age.

**Figure 2:**
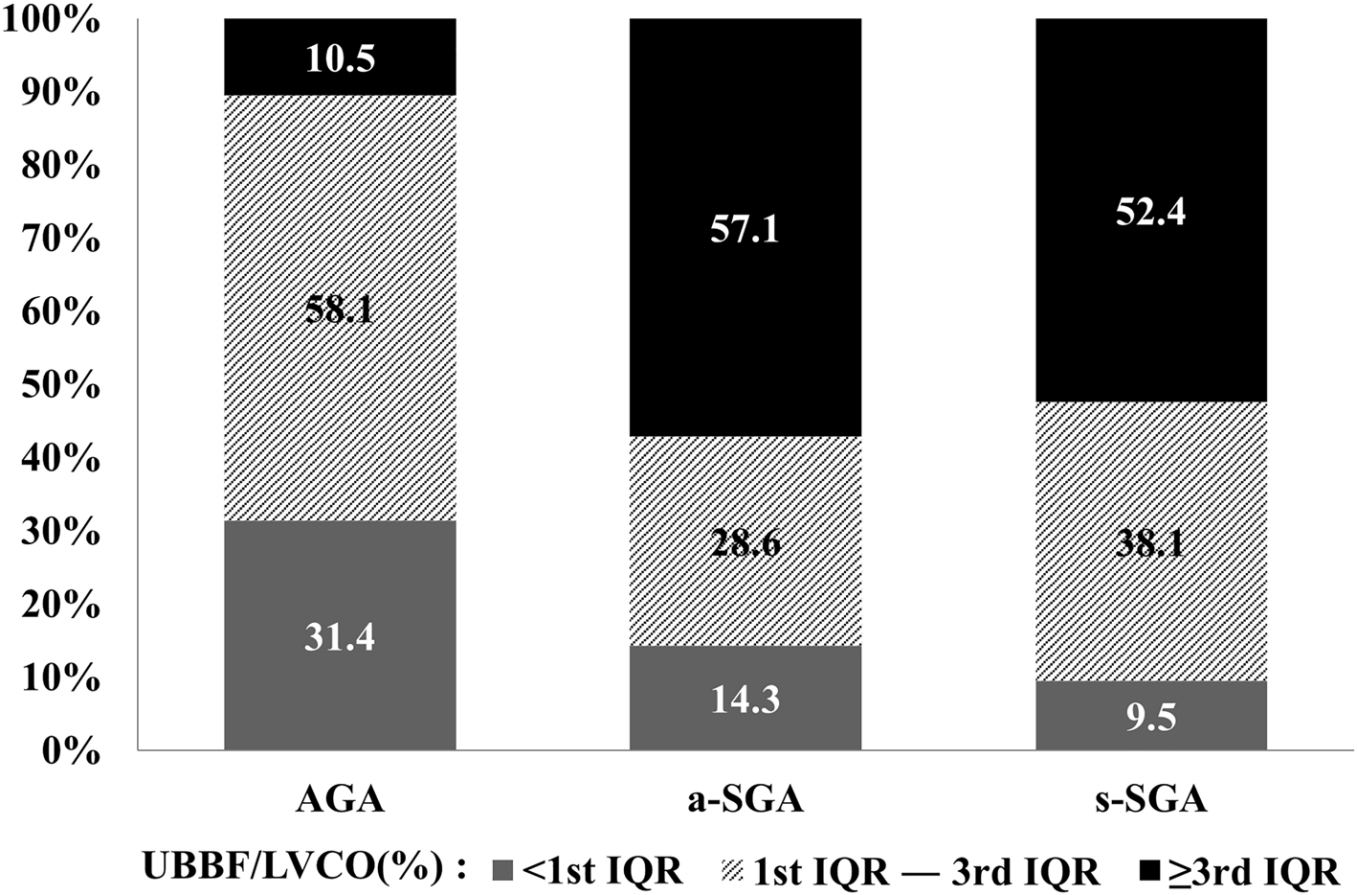
Distribution of UBBF/LVCO (%) in AGA, s-SGA, and a-SGA groups. Abbreviations *AGA* Appropriate for gestational age, *a-SGA* Asymmetric small-for-gestational age, *IQR* Interquartile range, *LVCO* Left ventricular cardiac output, *UBBF* Upper body blood flow, *s-SGA* Symmetric small-for-gestational age.

Figure 2 shows the distribution of UBBF/LVCO (%) in different growth pattern groups. The 1st interquartile range (IQR) of UBBF/LVCO in the included infants was 35.8%, while the median was 45.3%, and the 3rd IQR was 58.2%. Both the a-SGA and s-SGA groups predominantly consisted of infants whose UBBF/LVCO was greater than the 3rd IQR (58.2%), while in AGA infants, only 10.5% of infants presented with UBBF/LVCO exceeding the 3rd IQR.

The post-discharge neonatal outcomes were analyzed depending on the extent of brain sparing, represented by UBBF/LVCO (Table 4). Neonates with UBBF/LVCO ≥ 3rd IQR exhibited a significantly higher frequency of having at least one screen-positive field in the K-DST or receiving rehabilitative therapy later in the course of follow-up hospital visits. Additionally, head circumference < 10th percentile at a corrected age of 4–5 months was observed twice as frequently in this group, but this was not statistically significant.

**Table 4:** Post-discharge outcomes according to the UBBF/LVCO ratio (%).

	UBBF/LVCO < 1st IQR (n = 26))	UBBF/LVCO1st IQR ≤ and < 3rd IQR (n = 58)	UBBF/LVCO ≥ 3rd IQR (n = 26)	*P*
HC < 10th percentile	3 (11.5)	6 (10.3)	6 (23.1)	NS
K-DST < − 2SD	7 (26.9)	11 (18.6)	11 (42.3)	0.024†
Rehabilitative therapy	5 (20.0)	17 (28.8)	16 (61.5)	0.011‡

^†^Significant after Bonferroni correction (1st-3rd IQR vs. ≥ 3rd IQR).

^‡^Significant after Bonferroni correction (< 1st IQR vs. ≥ 3rd IQR, 1st-3rd IQR vs. ≥ 3rd IQR).

*HC* Head circumference, *IQR* interquartile range, *LVCO* Left ventricular cardiac output, *SD* Standard deviation, *UBBF* Upper body blood flow.

**Figure 3:**
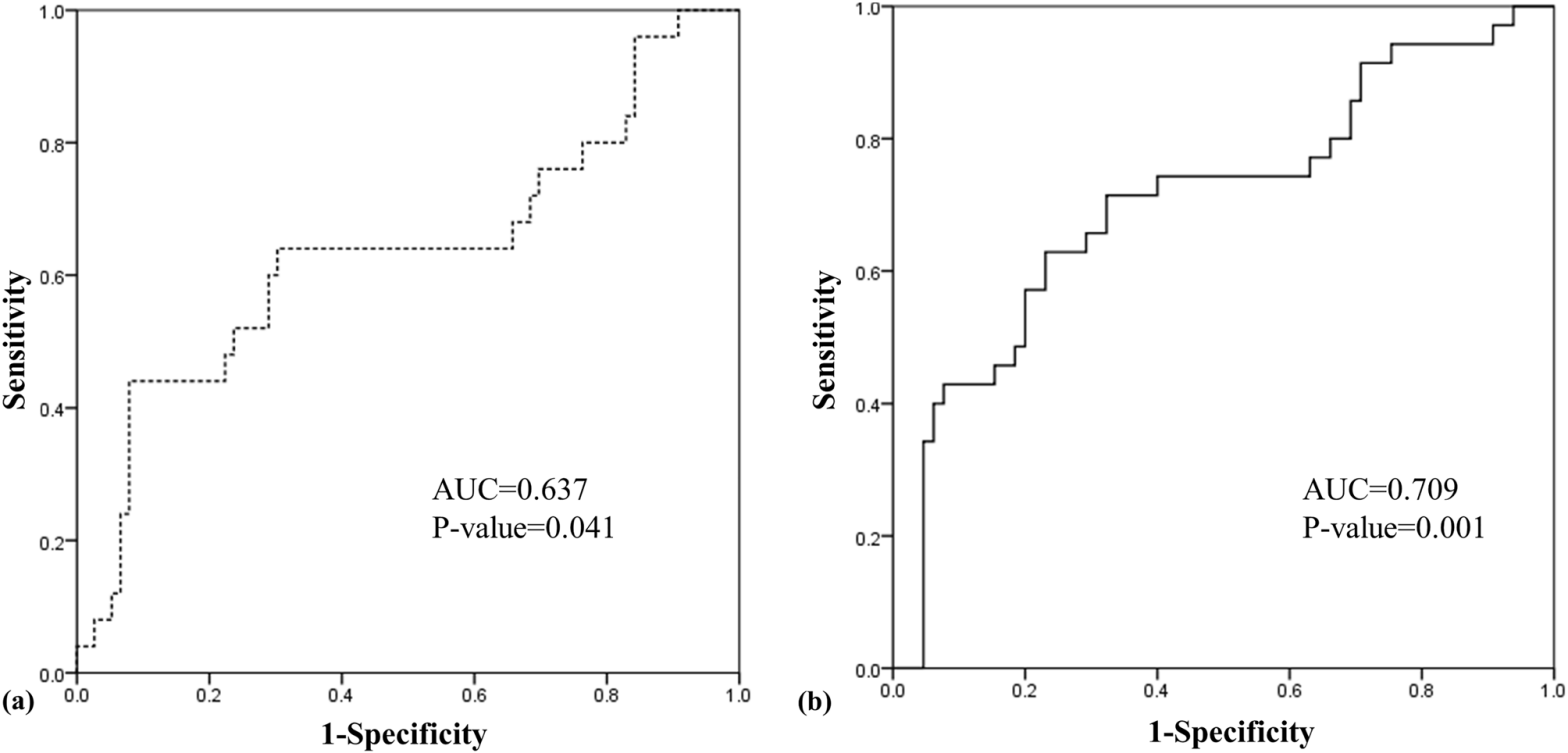
ROC curves of UBBP/LVCO (%) for evaluating association with adverse post-discharge outcomes (a: <  − 2SD in at least 1 field of K-DST, b: Later rehabilitative therapy). Abbreviations *ROC* Receiver operating characteristic, *AUC* Area under the curve, *K-DST* Korean developmental screening test for infants and toddlers, *LVCO* Left ventricle cardiac output, *SD* Standard deviation, *UBBF* Upper body blood flow.

The association between UBBF/LVCO and later adverse outcomes was analyzed using ROC curves (Fig. 3). The area under the curve (AUC) showed a fair value of 0.709 for the association with rehabilitative therapy at any time after discharge (*p* = 0.001), while the UBBF/LVCO AUC for the association with screen-positive results for K-DST was 0.637. Thus, predictivity analysis was performed only for rehabilitative therapy during follow-up; when the cut-off value was set to UBBF/LVCO ≥ 58.2%, the predictivity of receiving rehabilitative therapy showed 42.1% sensitivity and 86.1% specificity with a positive predictive value of 61.5%, negative predictive value of 73.8%, odds ratio of 4.5, and risk ratio of 2.4.

## Discussion

We assessed the course of the brain-sparing effect during early postnatal cardiovascular adaptation in growth-restricted infants in comparison with AGA infants using echocardiographic parameters. Based on our study findings, the brain-sparing effect represented by UBBF/LVCO (%) seemed to continue in some infants with a cutoff value of ≥ 58.2%, UBBF/LVCO was associated with a later need for rehabilitative therapy after discharge.

Although there are many etiologies leading to SGA, including placental insufficiency, congenital infection, chromosomal anomaly, or constitutionally small birthweight, SGA^20^ is often used as a proxy for FGR as it alerts clinicians to the possibility of chronic intrauterine hypoperfusion and hypoxia. Maternal hypertension is one of the factors contributing to placental insufficiency; because infants with documented congenital infection of chromosomal/genetic anomaly were excluded, the higher prevalence in s-SGA infants compared to AGA or a-SGA infants may imply the chronicity and severity of hypoxia resulting in small head circumferences in s-SGA infants.

In our study, we used early postnatal echocardiography to assess UBBF/LVCO, which reflects cerebral blood flow. Brain sparing in response to chronic intrauterine hypoxia not only alters the structure and function of the cerebral vasculature to increase cerebral blood flow but also increases placental vascular resistance^3^ and right ventricular afterload as a result of peripheral vascular bed vasoconstriction^21^. Such cardiac remodeling leads to an increase and decrease in the preload and afterload in the left ventricle, respectively. The trend of this effect during the early postnatal days has been investigated in some previous studies. In one study, LVCO on the first day of life was significantly greater in SGA infants versus AGA infants (243 ± 47 versus 150 ± 28 mL/kg/min); however, according to sequential assessments, LVCO increased by 89% in AGA infants, while that in SGA infants remained similar^22^. Fouzas et al.^23^ demonstrated a similar study result, in which a decrease in heart rate and an increase in LVSV and LVCO from the second to fifth postnatal day were observed in AGA infants, while in growth-restricted infants, although the heart rate was significantly higher compared to the AGA infants, stroke volume showed no significant change, and LVCO even decreased throughout the assessment time points. Blood pressure sequentially increased in AGA infants, contributing to the loss of significant difference compared to growth-restricted infants, who had higher values on the second postnatal day. This implies that growth-restricted infants have a limited myocardial reserve at birth, which is compensated for by increasing the heart rate to preserve cardiac output. In the current study, the median (interquartile range) LVSV and LVCO were significantly greater in both a-SGA and s-SGA infants than in AGA infants [224 (196–259) *vs* 271 (217–298) *vs* 186 (151–210) mL/kg/min, respectively], at a median 2 to 3 days of life. Heart rate was higher in SGA infants than in AGA infants, but the difference was not statistically significant, and blood pressure was also similar among the three groups. However, it is possible that these findings were affected by the limited number and various disease statuses of enrolled infants.

The a-SGA and s-SGA infants presented similar UBBF/LVCO in the early postnatal days despite significant differences in head circumference measurements at birth. The head circumference measurements at 4–5 months corrected age were approximately 1 cm smaller in both SGA groups than in the AGA group. The prevalence of head circumference < 10th percentile at 4–5 months corrected age was more than twice as high (23.1% *vs*. 11.3% and 10.3%, respectively) in the UBBF/LVCO ≥ 3rd IQR group when compared to that of the other two groups with lower UBBF/LVCO. Although the statistical insignificance of head circumference differences at post-discharge may have been the result of the small sample size, these findings may imply that altered cerebral hemodynamics and degree of blood flow redistribution may not necessarily be associated with head circumference at birth in growth-restricted infants, but the effect of brain sparing may impact postnatal head growth in the long run. However, large-scale follow-up studies are required for verification.

A UBBF/LVCO cutoff value of ≥ 58.2% was associated with adverse neurodevelopmental outcomes in our study. Recent studies have shown contradictory results concerning the protective effects of brain-sparing. Richter et al.^24^ reported that fetal brain sparing (defined as CPR < 1) did not show a significant association with the intellectual quotient in growth-restricted infants at the age of 4, while better outcomes in terms of total behavior and externalizing behavior were observed in those with brain sparing. In a study by Bellido-Gonzalez et al.^25^, various parameters for brain sparing were used, including MCA PI (< 5th percentile), CPR (< 5th percentile), and uterine artery PI (≥ 95th percentile). In their study, when brain sparing was defined as satisfying all three parameters, growth-restricted infants had a significantly lower full-scale intellectual quotient at ages 6–8 compared to AGA infants, but when the latter two parameters were normal, the outcome between the two groups of infants was similar. The association between brain sparing and neurodevelopmental performance at 40 weeks’ corrected age has also been studied ^7,26^. Preterm SGA infants with brain sparing (defined by MCA PI < 5th percentile) in comparison to AGA infants presented significantly lower scores in the fields of habituation, motor, social interaction, and attention in the neonatal behavioral assessment scale. However, when such assessments were performed in term infants, the results were variable. The reason for such contradictory results reported in previous literature is rooted in the use of different criteria to define brain sparing. In addition, although MCA PI is one of the most frequently used parameters to define brain sparing, the blood flow change in the ACA precedes that in the MCA^27^, which raises the possibility of misclassifying infants who have already been affected by brain sparing that has not yet been recognized.

Our study has some limitations. As this was a retrospective study with a small sample size in a single institute, we could not perform a multivariable analysis. However, we attempted to partially compensate for this disadvantage by matching several important factors in recruiting AGA infants for the control group. Nonetheless, because completely normal infants usually do not undergo routine echocardiographic assessment, it was impossible to include infants without any clinical problems in this study. This may have led to some differences in the clinical characteristics of the included infants that were not readily obvious in their medical records, which is another shortcoming of our study. Therefore a future study with a completely ‘clean’ sample as the control group is needed to verify our findings. Furthermore, although SGA and FGR have been previously used interchangeably, they are not identical. As recent definition of FGR^28^ encompasses abnormal intrauterine vascular Doppler findings in addition to weight percentile criteria, and also faltering intrauterine growth, constitutional SGA infants or growth-restricted AGA infants may be falsely classified as FGR and non-FGR. Furthermore, the onset of FGR (early vs late) contributes to different phenotypes and may be a major factor impacting fetal and neonatal outcomes of the affected infant. Since the information on intrauterine markers^28^ of fetal growth retardation and signs of malnutrition on postnatal physical examination^29^ were not fully available, we used SGA, the simplest proxy for FGR. It should be kept in mind that our definition may have led to a selection bias. Nevertheless, although placental diseases and their impact in late FGR infants may be of a lesser extent, late-onset placental dysfunction in a portion of late FGR^30,31^ and the association of placental insufficiency in both types of FGR have been discussed in the literature^32^. Also, brain sparing represented by advanced brain vasodilation suggesting chronic hypoxia in 25% of late FGR is possible^33^. Moreover, since a non-negligible portion of infants with late FGR go unnoticed^34^, our study may have a role in monitoring, alerting, and supporting patient treatment decisions in clinical settings.

In addition, echocardiographic measurements have a few limitations. Because the CSA is squared from the diameter, measurement errors can be multiplied. There is also an inevitable risk of error when manually tracing the PW Doppler to obtain VTI^17^. However, only one person reviewed and obtained echocardiographic measurements using the above-mentioned program, which ensured consistency in measurement. The reliability of the measurement of blood flow using echocardiography has been well described in the literature, showing strong correlations with other methods such as phase-contrast MRI, pressure measurement using a cardiac catheter, and Fick’s dye dilution method^14,35,36^. Although the measured UBBF may be higher in cases of ductal patency, PDA in preterm infants > 30 weeks’ gestation show a spontaneous closure rate of 90% and 98% at day 4 and day 7 of life, respectively^37^. In addition to the examination time frame of 7th postnatal day in our study, we excluded infants with hemodynamically significant PDA. Thus, our results are expected to be minimally impacted. Of note, previous studies^38^ report persistent alterations of cerebral and cardiac hemodynamical changes in SGA/FGR infants during variable postnatal periods ranging from less than a week to even after the second week of life. We decided on the time frame of 7 days to limit the influence of possible PDA shunt during the earliest days of life and at the same time minimize the confounding effects of prolonged disease status and medications.

Finally, we used the K-DST, a screening tool to assess the neurodevelopmental outcomes of the included infants. The infants admitted to the NICU were not subject to the same follow-up protocols. The more severe the disease status, the longer the set follow-up schedules, and more definitive assessment tools such as the Bayley Scales of Infant and Toddler Development-III were used. Because very preterm and critical infants who were hemodynamically compensated were excluded, long-term follow-up visits (as long as the corrected age of 18 months or chronological age of 3 years, as per the high-risk infants’ follow-up schedules in our institute) and the use of more rigorous tests were limited.

Even considering these limitations, this study provides the following contributions. To the best of our knowledge, this study is the first to suggest the usefulness of UBBF/LVCO as the surrogate of brain sparing in neonates. As mentioned previously, the most common criterion for fetal brain sparing is MCA PI with a cutoff value of < 5th percentile. CPR, which is yielded by dividing MCA PI by uterine artery PI, is also used^8^. Blood flow velocities, PIs of MCA or ACA^9^, or near-infrared spectroscopy have been used to predict cerebral blood volume^10,11^ in early postnatal life. However, the accuracy of these measurements is questionable because blood flow velocity is affected by viscosity, vessel diameter, and vessel wall resistance, leading to contradictory results across studies^39^. In contrast, the parameters used in our study fundamentally incorporate the volume measurement obtained via calculations involving blood flow velocity, CSA, and time integrals in large cylindrical vessels, where the results of the calculations would be less likely affected by the blood viscosity and vessel wall resistance compared to smaller vessels like MCA or ACA. Meanwhile, superior vena cava flow, another surrogate marker of cerebral blood flow, is D-shaped and the diameter variation according to breathing is severe, which makes errors in CSA measurement more likely to occur than relatively non-collapsible arteries^17^. In this regard, UBBF/LVCO (%) may have merits as a novel surrogate of brain sparing. Furthermore, postnatal echocardiographic assessment of brain sparing can be useful when information from antenatal assessments is lacking. In addition, as much as 25% of late FGR infants go unnoticed until or shortly before delivery, thus exposing some of the affected infants to rapid placental function failure or intrapartum fetal distress^32^. Identifying the echocardiographic findings suggestive of maintained brain-sparing effect in SGA infants during early postnatal life may alert the clinicians to further investigate for clues of chronic intrauterine hypoxia and guide follow-up plans for infants considered at-risk. For instance, infants with UBBF/LVCO of approximately 60% or more may be at risk for adverse neurodevelopmental outcomes in the future based on our study findings; therefore, a more detailed medical inspection may be necessary to distinguish the infants who may benefit from early intervention to improve neurodevelopmental outcomes. Future research is warranted to prospectively investigate the duration of brain sparing after birth, long-term neonatal outcomes according to the presence and changes of brain sparing in a larger population, and the potential applicability of these findings to premature infants.

## Conclusion

Based on our study, the brain-sparing effect seems to continue during early postnatal life in SGA infants, and such findings may be a clue, warranting further investigation to assess the possibility of future adverse neurodevelopmental outcomes. Blood flow distribution assessed by echocardiography is a promising tool, particularly when antenatal assessment information is lacking. However, our study findings need further validation before they are extended to clinical monitoring and decision-making. Future larger-scale, prospective studies are needed to verify our findings and extend the clinical usefulness of echocardiographic assessment of brain-sparing.

## Data Availability

The data is available only upon a reasonable request to the corresponding author.
